# Evaluation of Surface Roughness by Image Processing of a Shot-Peened, TIG-Welded Aluminum 6061-T6 Alloy: An Experimental Case Study

**DOI:** 10.3390/ma11050771

**Published:** 2018-05-10

**Authors:** Anas M. Atieh, Nathir A. Rawashdeh, Abdulaziz N. AlHazaa

**Affiliations:** 1 Industrial Engineering Department, School of Applied Technical Sciences, German Jordanian University (GJU), Amman 11180, Jordan; 2 Mechatronics Engineering Department, School of Applied Technical Sciences, German Jordanian University (GJU), Amman 11180, Jordan; Nathir.Rawashdeh@gju.edu.jo; 3 Department of Physics & Astronomy, College of Science, King Saud University, Riyadh 11451, Saudi Arabia; alhazaa@gmail.com; 4 King Abdullah Institute for Nanotechnology (KAIN), King Saud University, Riyadh 11451, Saudi Arabia

**Keywords:** materials characterization, shot-peening, image processing, TIG welding, aluminum 6061-T6

## Abstract

Visual inspection through image processing of welding and shot-peened surfaces is necessary to overcome equipment limitations, avoid measurement errors, and accelerate processing to gain certain surface properties such as surface roughness. Therefore, it is important to design an algorithm to quantify surface properties, which enables us to overcome the aforementioned limitations. In this study, a proposed systematic algorithm is utilized to generate and compare the surface roughness of Tungsten Inert Gas (TIG) welded aluminum 6061-T6 alloy treated by two levels of shot-peening, high-intensity and low-intensity. This project is industrial in nature, and the proposed solution was originally requested by local industry to overcome equipment capabilities and limitations. In particular, surface roughness measurements are usually only possible on flat surfaces but not on other areas treated by shot-peening after welding, as in the heat-affected zone and weld beads. Therefore, those critical areas are outside of the measurement limitations. Using the proposed technique, the surface roughness measurements were possible to obtain for weld beads, high-intensity and low-intensity shot-peened surfaces. In addition, a 3D surface topography was generated and dimple size distributions were calculated for the three tested scenarios: control sample (TIG-welded only), high-intensity shot-peened, and low-intensity shot-peened TIG-welded Al6065-T6 samples. Finally, cross-sectional hardness profiles were measured for the three scenarios; in all scenarios, lower hardness measurements were obtained compared to the base metal alloy in the heat-affected zone and in the weld beads even after shot-peening treatments.

## 1. Introduction

In the past two decades, owing to the good mechanical properties, i.e., high strength to weight ratio, good thermal and electrical conductivity, aluminum and its alloys have been used in versatile engineering applications such as marine vessels, automobiles, railway cars, and aircraft [1,2,3,4,5]. 

Aluminum is strengthened through precipitation hardening (for instant the 6000 aluminum series) due to the presence of silicon and magnesium alloying elements (0.3–15 wt % Si and Mg). The addition of those alloying elements resulted in further developments of Aluminum characteristics, which include good formability, corrosion resistance, and weldability [6]. Aluminum 6061 has been widely used in aerospace applications, especially with the T6 tempered solution-heat-treated and artificially aged status [7,8].

Of the different welding techniques, Tungsten Inert Gas (TIG) welding, an arc welding process that uses a non-consumable electrode, has gained more attention in welding aerospace alloys, i.e., Al6061-T6. TIG welding quality was considered to be the most effective technique not only because of the high strength but also due to the cleanliness of the resulting weld bead with minimal defects produced [9].

One of aluminum’s drawbacks is the low wear resistance; furthermore, welded aluminum alloys are usually characterized by sever metallurgical changes within the heat-affected zone and grain coarsening in the adjacent base metal alloy [9,10,11]. Therefore, a surface treatment process is required to maintain the good mechanical characteristics of aluminum alloys. Moreover, because of the different performance and surface property requirements of aluminum and its alloys, different surface treatments could be done including surface mechanical attrition treatment [12,13,14], ultrasonic shot-peening [15,16], laser shot-peening [17,18,19,20], and air-blast shot-peening [19,21,22]. Among them, air-blast shot-peening has received increased attention. This process is a cold-working surface treatment, where a large amount of small spherical particles (called shots) are bombarded onto a metallic surface at high velocity [23,24,25].

In shot-peening, only a very thin surface layer is affected by the impact of shots, which produce tensile plastic strain, resulting in favorable compressive residual stresses. Those residual stresses are approximately equal to the process-induced stress and could be estimated and calculated analytically [25]. However, shot-peening must be optimized for specific materials and specific conditions, i.e., welded material; otherwise, it may have undesirable results such as surface contamination, reduction in strength, and surface crack formation [26,27,28].

Recently, many researchers have investigated the effect of different shot-peening techniques on the weldment’s characteristics [16,17,29,30]. Yang et al. studied the shot-peening effects on dovetail specimens’ joints of Ti-6Al-4V fretting fatigue behavior. It was reported that shot-peening altered the crack initiation mechanisms and enhanced the fretting fatigue performance for the titanium alloy [31]. Dissimilar TIG-welded joints of magnesium and titanium alloys were also subjected to high-energy shot-peening and the microstructure and mechanical properties in terms of tensile strength were evaluated by Chuan Xu et al. [32]. Surface defect elimination, strengthening by grain refinement, and strain hardening were a result of subjecting Mg/Ti welded joints to high-energy shot-peening. Furthermore, the tensile strength of Mg/Ti TIG-welded samples was increased by 24.5% to 241 MPa [32].

Different researchers have been utilizing different image processing techniques and real-time measurements. The main focus was to capture the properties and characteristics of weldments and to anticipate the quality of weld beads during live welding processes [33,34,35,36,37,38]; however, the authors are not aware of any report on the application of image processing in the detection of surface properties of welded and shot-peened alloys. The image processing techniques have been utilized to characterize different material properties [39,40,41,42,43,44,45,46]. For example, external welding defects have been widely inspected by vision-based and image processing techniques [41,43,47].

In this work, we present experimental results from a post-TIG-welded shot-peened Al6061-T6 alloy with such an analysis approach. Image processing algorithms have been utilized to extract information from the acquired images of the samples after surface processing to evaluate the surface roughness parameters. Using the proposed technique, it is expected to accelerate obtaining the characterization of the surface properties of the welded samples compared to traditional destructive testing. This may also enhance the detection reliability and overcome the traditional measurement limitations of weldment characteristics. The novel contribution of this research work is the development of an image processing technique that can measure the crater/dimple size of the post-weld shot-peened Al 6061-T6 alloy. Furthermore, we reconstruct the 3D weld surface morphology accurately and reliably for monitoring and evaluating post-process surface properties to be utilized in industrial applications.

## 2. Methodology

### 2.1. Materials, Properties, and Testing Specimens

The materials used in this study are aluminum alloy Al 6061-T6 supplied by Jordan Airmotive Company-JALCO from ALCOA Inc., Davenport, IA, USA. Raw materials were provided in sheet metal format with chemical composition as in Table 1.

**Figure 1:**
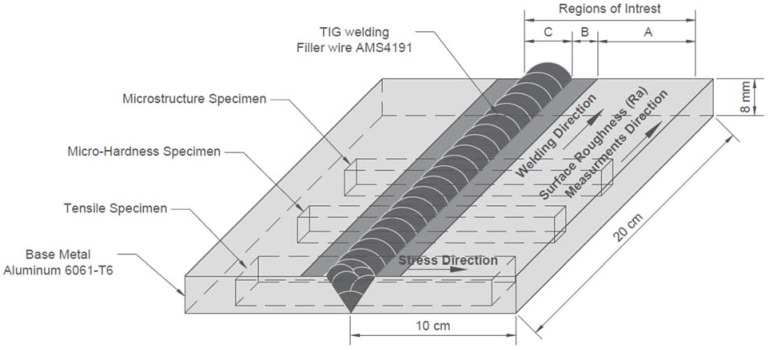
Geometry of TIG-welded Al6061-T6 specimen and schematic of testing specimens showing welding and surface roughness measurement direction.

This solution and precipitation heat-treated lightly oil coiled sheet aluminum alloy was produced according to aerospace materials specifications AMS4027N (ASTM B209-14). Rectangular specimens of 10 cm × 20 cm and 8 mm thickness were cut and then butt form welded according to AWS17.1 standard using filler wire of AMS4191. The TIG welding was carried out under 99.99% pure argon shielding gas and 72 amps AC welding current. In addition to the control sample (only TIG-welded, see Figure 1), two other sets of TIG-welded samples were prepared for shot-peening. The welded samples were subjected to two shot-peening scenarios after the welding: high-intensity and low-intensity shot-peening. The controlled shot-peening treatment was performed on the Al6061-T6 alloy by means of an E-S-1580 Pangborn-controlled shot-peening machine.

**Figure 2:**
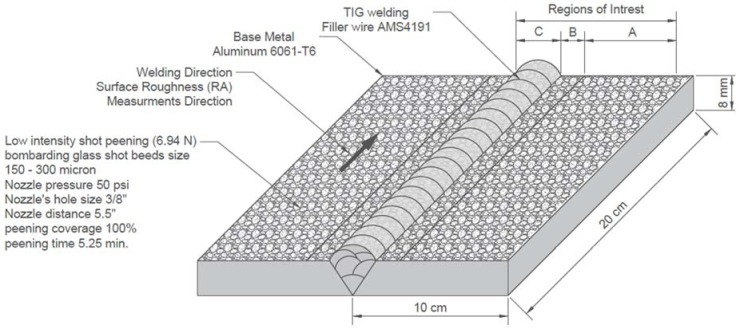
Geometry of Al6061-T6 low-intensity shot-peened, TIG-welded specimen.

The low-intensity shot-peening (6.94 N) was produced by bombarding glass shot beads. The glass shot beads were aerospace controlled level (OMAT 1/239 glass beads) with a grit number ranging from 150 to 300/RR and the size of glass beads ranged from 150 to 300 microns. This conforms to Rolls Royce CSS8 issue 5 styker orthopedics MS00097 issue 4, under a nozzle pressure of 50 Psi with a 3/8-inch nozzle size hole and a 5.5-inch nozzle distance. The low-intensity shot-peening lasted for 5.25 min, and the peening coverage was 100%, as confirmed by an optical magnifier (see Figure 2).

**Figure 3:**
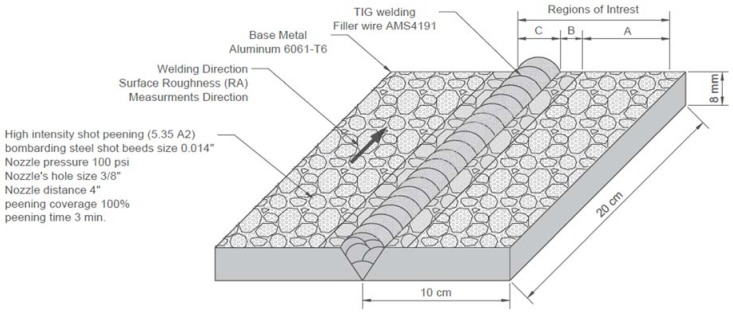
Geometry of Al6061-T6 high-intensity shot-peened, TIG-welded specimen.

As for the high-intensity shot-peening (5.35 A2), it was produced by bombarding steel shot beads. The steel shot beads were aerospace controlled level, spherically condition wire cut with 0.014-inch size (SCCW-0.014-inch) under nozzle pressure of 100 Psi with 3/8-inch nozzle size hole and 4-inch nozzle distance. The high-intensity shot-peening lasted for 3 min, and the peening coverage was 100%, as confirmed by an optical magnifier (see Figure 3).

At both intensities, the Almen gauge #2 model TSP-3 Rev. B were used with different Almen strips to achieve the required intensities by subjecting the strips to peening at different table speeds until the required saturation point (intensity) was achieved. 

### 2.2. Characterization 

**Figure 4:**
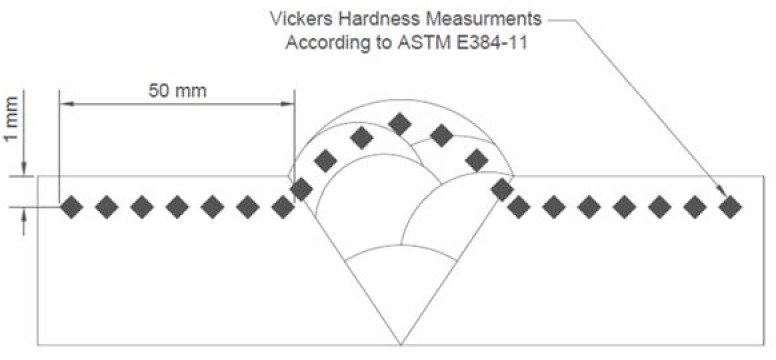
Micro-hardness measurement profile.

Vickers hardness measurements were performed on shot-peened treated samples at both intensities along with the control samples. Measurements were performed according to ASTM E384-11^ɛ1^ standard on a Tru-Blue united hardness tester (Tru-Blue U/10 version F13, San Diego, CA, USA) by applying a 10 kg load for 15 s dwell time. Four measurement sets were taken on the cross section of the mounted samples, 1 mm away from the shot-peened surface (see Figure 4).

Surface roughness (Ra) SJ-210 Mitutoyo Japan tester (Mitutoyo, Kawasaki City, Japan) was used according to ISO11562 standard, where measurements were taken parallel to the weld bead’s in the base metal alloy region only. Roughness on the weld-line and on the heat-affected zone was very rough and out of device measurement capabilities. The roughness readings’ sampling length was 8 mm and the evaluation length was 40 mm at a constant reading speed of 0.5 mm/s (see Figure 1). The microstructure and tensile specimens obtained from those samples were kept for further analysis in stage 2 of this project.

### 2.3. Image Processing 

An optical microscope was used to capture 8-bit intensity Tagged Image File Format (TIFF) images at a resolution of 2067 pixels per millimeter. Three regions of interest (ROI) were identified, A, B, and C, with an area of 0.8 mm^2^. The microscope (Zeiss Discovery V20) (Carl Zeiss MicroImaging GmbH, Göttingen, Germany) was equipped with an LED ring-light around the microscope lens, and a digital camera (AxioCam ERc 5s) (Carl Zeiss MicroImaging GmbH, Göttingen, Germany) configured for 95X magnification. Then equivalent surface topography images were generated in which the light areas are shown as mountains and the dark areas as valleys. These images approximate the real topography. The goal is to compute optical metrics from the magnified surface texture image, for various material properties including hardness, roughness, and dimple size distribution. Hence, for comparative analysis, images were taken from identical regions A, B, and C as shown in Figure 1, Figure 2 and Figure 3.

## 3. Results and Discussion

**Figure 5:**
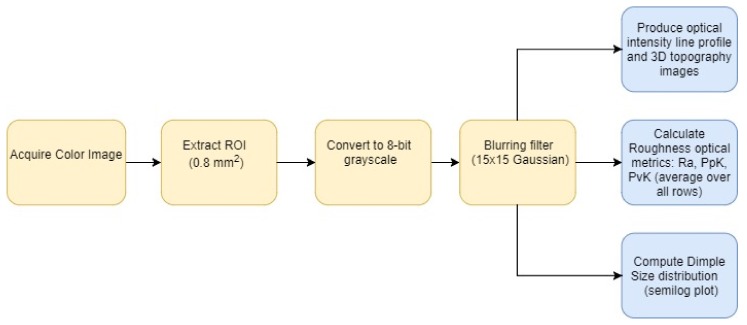
Image processing algorithm for analysis.

**Figure 6:**
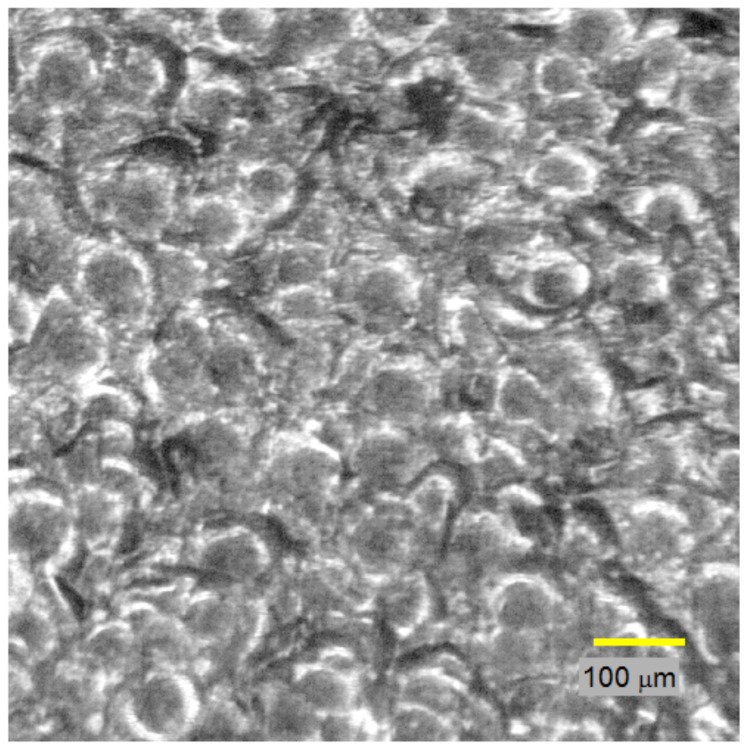
Example of a low-intensity shot-peened metal, of size 0.8 mm^2^, i.e., 1653 × 1653 pixels.

The image processing steps are summarized in the block diagram shown in Figure 5. The acquired color images are cropped to represent a square of 0.8 mm^2^ for all regions of interest. The region of interest (ROI) image is then converted to an 8-bit grayscale image where an intensity value of 0 represents black, and 255 represents white. The image is then blurred to produce smoother intensity histograms, i.e., more natural intensity level distribution, where all intensities are present. From there, it is possible to produce line profiles and approximate 3D topography images for qualitative comparisons. In addition, further processing produces optical roughness and hardness metrics that correlate with the measured data produced using specialized laboratory tools such as SJ-210 Mitutoyo for surface roughness and Tru-Blue U/10 for hardness. Lastly, a matched filter process on the 2D image produces dimple size distribution plots that can be used qualitatively to compare the roughness of imaged sample regions. More details about these steps are presented in the example shown in Figure 6.

**Figure 7:**
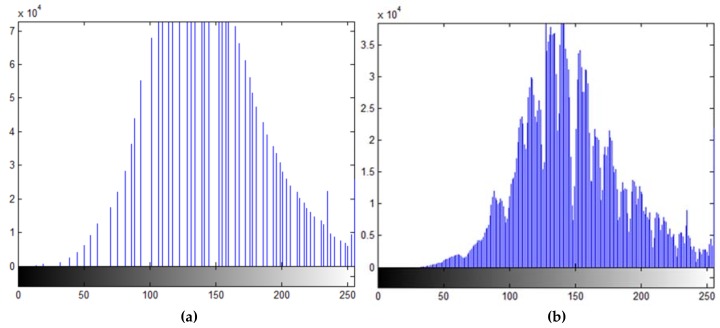
Intensity histograms of a low-intensity shot-peened sample: (**a**) before blurring; (**b**) after blurring.

Mostly, pixel-intensity image processing techniques are applied on the obtained microscope images to analyze various surface properties. The analysis starts by applying a slight blur to the image to enhance the intensity histogram detail. The blurring filter used a 15 × 15 Gaussian mask for convolution. The effect of blurring on the intensity histogram is shown in Figure 7. It can be seen that in the smoothed image histogram, the pre-blur histogram has empty intensity bins, which is attributed to the TIFF image format compression artifact. With the blurred image histogram, it is possible to choose intensity-level cutoffs to classify pixels as being of dark or medium intensity. 

**Figure 8:**
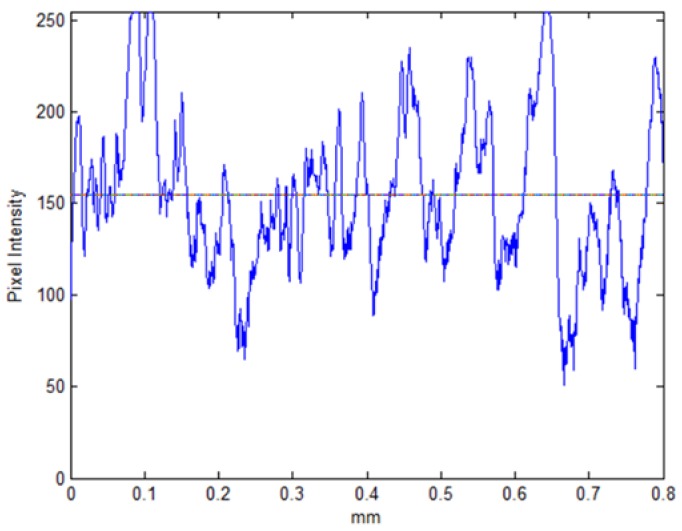
Optical intensity line profile of the center horizontal line in the example image. The average intensity level is depicted as a line.

**Figure 9:**
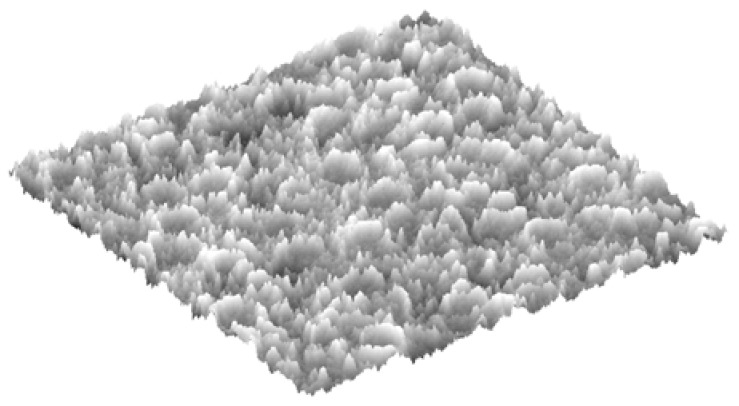
Optical approximation of the 3D topography of the example image of size 0.8 mm^2^.

In order to investigate the samples’ surface, it is helpful to generate an optical line profile of the ROI along with the average intensity on that line. Such a profile, shown in Figure 8, approximates the line topography, where high-intensity pixels represent elevated points and low-intensity pixels represent low elevation points. This shows the pixel intensities of a horizontal line through the center of the ROI, i.e., the center row of the example image in Figure 6. Similarly, an optical approximation of the 3D topography can be generated and used for qualitative analysis. This is shown in Figure 9 for the same example in the image presented in Figure 6. 

The optical line profile is used to calculate roughness metrics to overcome the measurement limitations using laboratory instruments. This imaging approach has the advantage of calculating the roughness metrics as an average of all line profiles in the ROI, i.e., all horizontal lines starting at the top and ending at the bottom, of which there are *J* = 1653. In order to define the equations for this procedure, the first step is to describe the optical roughness profile as containing *I* ordered, equally spaced points along the trace, and defining *y**_i_*** as the vertical distance from the mean line to the *i*th data point. Height is assumed to be positive in the up direction, away from the bulk material. The following equations define the roughness metrics, Roughness Average *Ra*, Max Peak Height *P_p_K*, and Max Valley Depth *P_v_K*:

(1) $$R_{a}=\frac{1}{J}\overset{J}{\underset{j=1}{\sum }}\left(\frac{1}{I}\;\overset{I}{\underset{i=1}{\sum }}\left|y_{ij}\right|\right)$$

(2) $$P_{P}K=\frac{1}{J}\overset{J}{\underset{j=1}{\sum }}\left(\max\left[y_{ij}\right]\right)$$

(3) $$P_{v}K=\frac{1}{J}\overset{J}{\underset{j=1}{\sum }}\left(\min\left[y_{ij}\right]\right),$$

where *j* is the image row index, i.e., the current line profile number, and *y_ij_* is the vertical distance from the mean profile intensity of the pixel at row *j* and column *i*. 

The final metric values are represented as a percentage of the 8-bit intensity range from 0 to 255 as follows:

Final *R_a_* = (*R_a_* / 255) ×100 (4)

Final *P_p_K* = (*P_p_K* / 255) ×100 (5)

Final *P_v_K* = (*P_v_K* / 255) ×100. (6)

**Figure 10:**
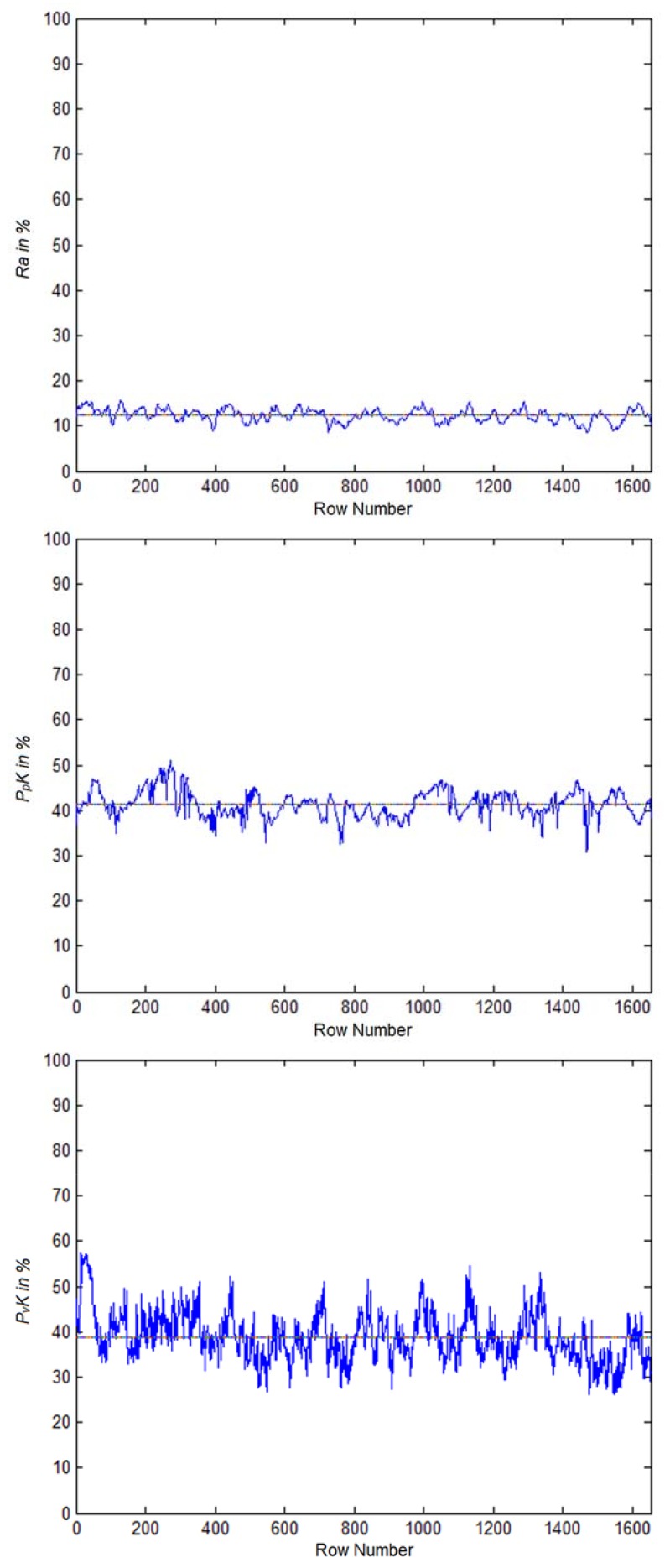
Optical roughness metrics versus ROI row number for the example image shown in Figure 6.

Figure 10 shows these metric values across ROI row number *j*. These intermediate plots illustrate the variability of the metrics over line profiles from top to bottom. The final metrics are averages of this data and deliver roughness estimates across the whole ROI rather than a few selected lines as in the case of laboratory equipment measurements.

**Figure 11:**
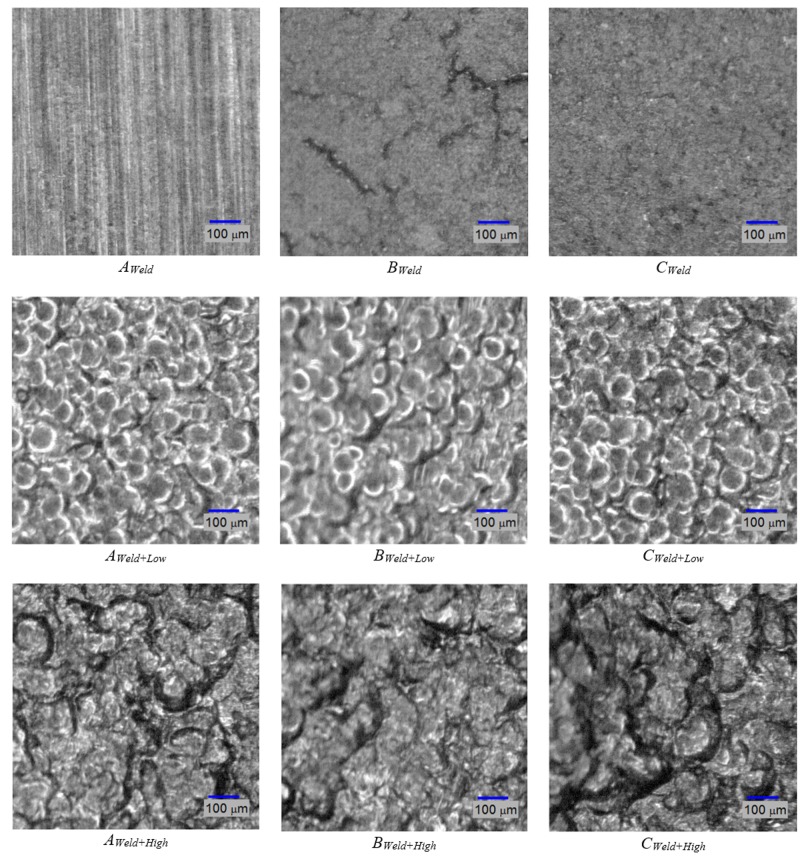
Grayscale images of three samples with three regions each.

**Figure 12:**
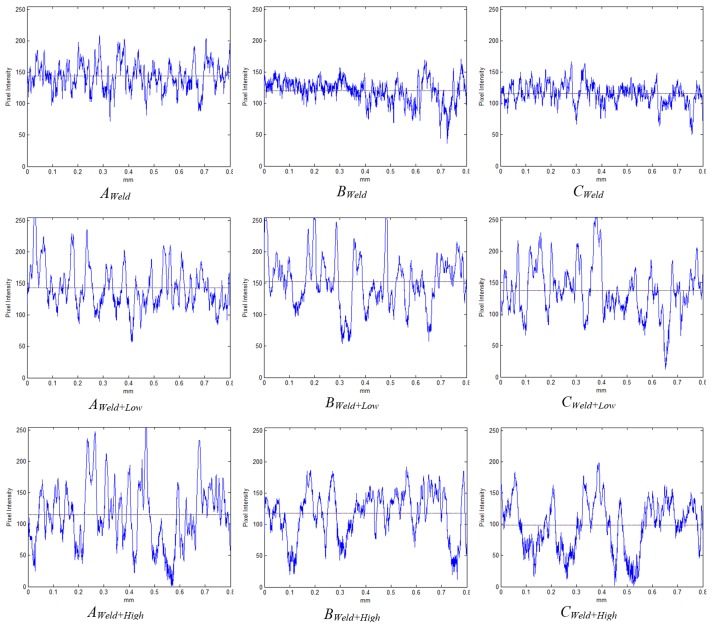
Line profile (center row) for three samples with three regions each. Length = 0.8 mm. Intensity is 0 for black and 255 for white pixels.

**Figure 13:**
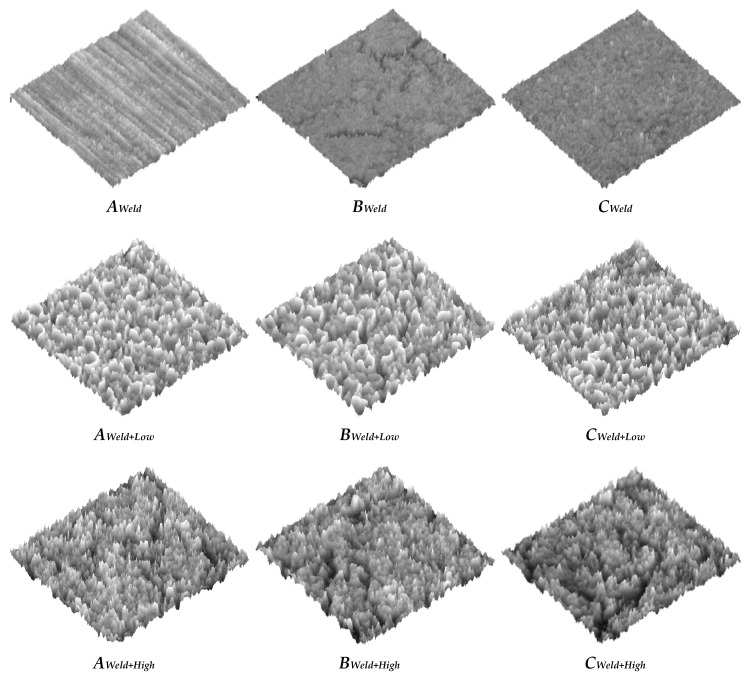
Surface topography images for three samples with three regions each, Image size = 0.8 mm^2^.

**Figure 14:**
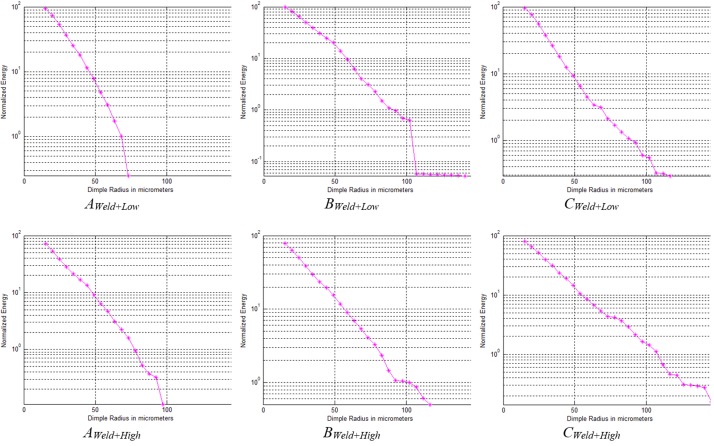
Dimple size distribution for two shot-peened samples with high and low intensity and three regions each.

**Figure 15:**
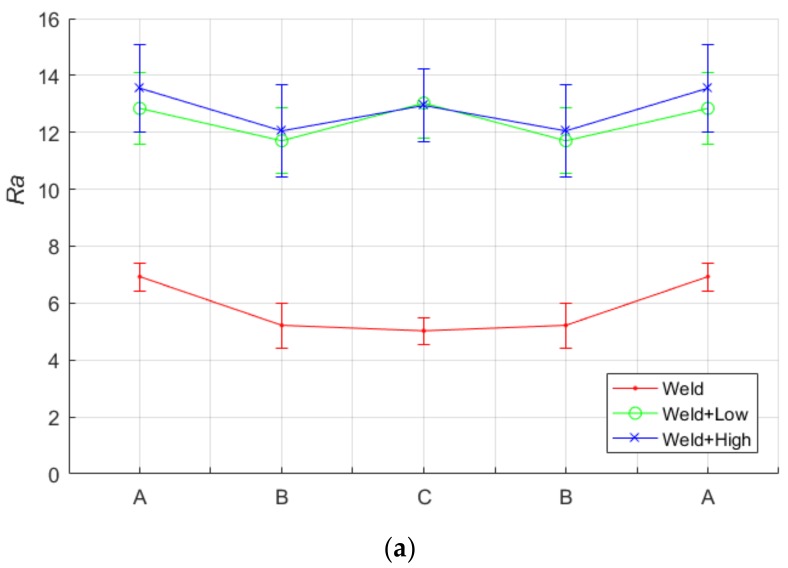

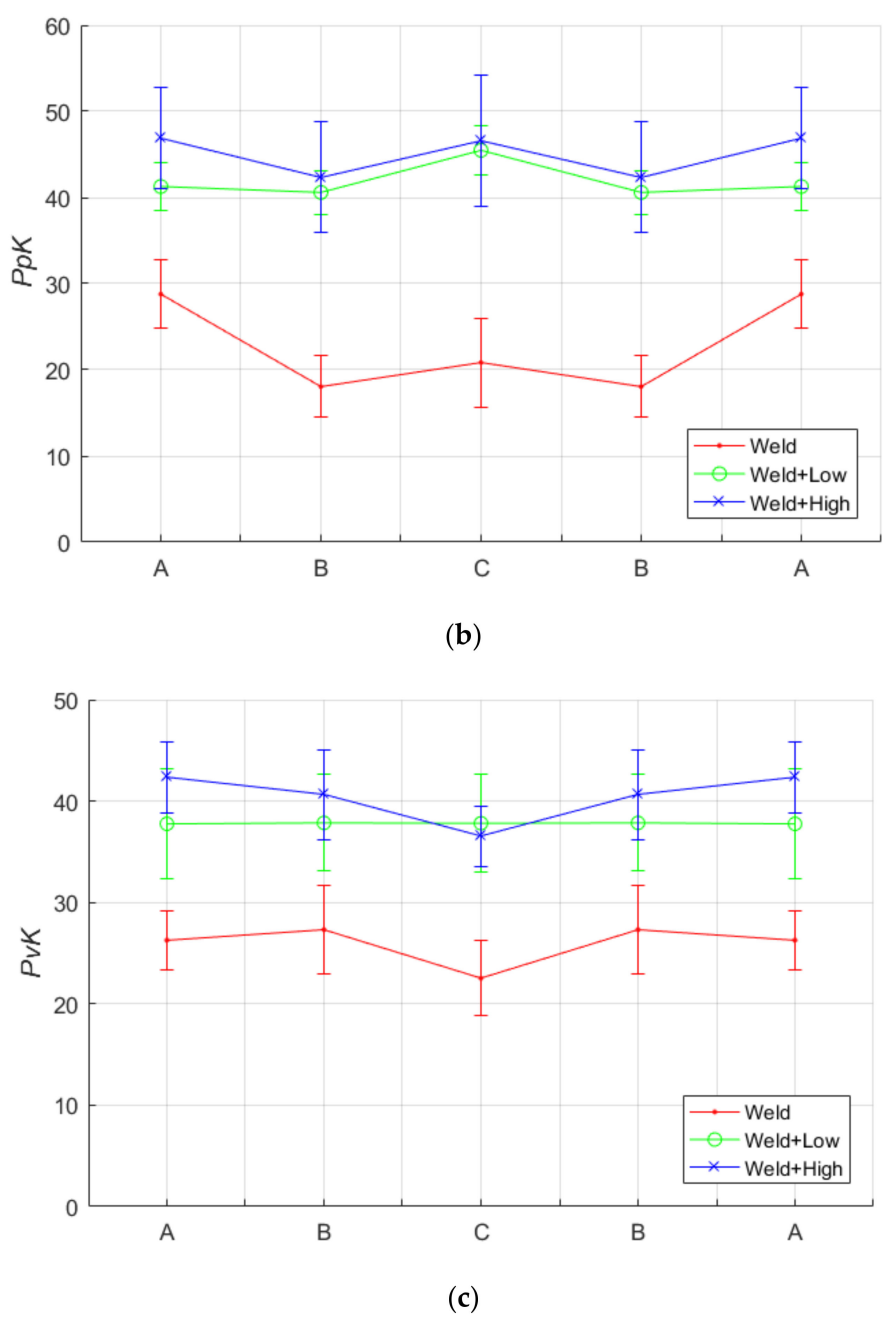
Optical roughness metrics (in %) for three samples in the three ROIs shown in Figure 11. (**a**) *R_a_*; (**b**) *P_p_K*; (**c**) *P_v_K.*

**Figure 16:**
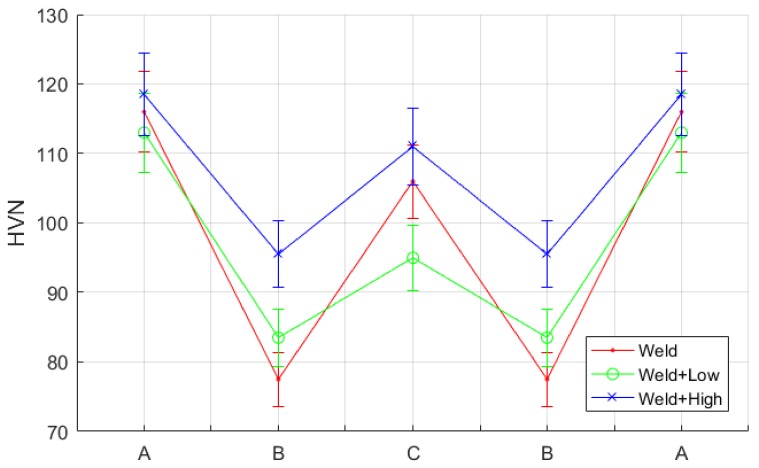
Hardness profile measurements for welded and shot-peened samples for the three regions of interest in Figure 11.

To validate the method of optically obtaining roughness measurements, three samples were chosen: A welded sample without shot-peening; a welded, low-intensity shot-peened sample; and a welded, high-intensity shot-peened sample. The ROIs for these samples are shown in Figure 11, and three regions for each sample are defined as in Figure 2. These regions are *A* (Base metal alloy), *B* (the heat affected zone), and *C* (the weld beads). Figure 12, Figure 13, Figure 14, Figure 15 and Figure 16 are all based on these selected samples’ ROIs. 

As for Figure 11 and Figure 12, they show that the welded sample (top row) is the least rough. The heat-affected zone, *B_weld_*, of this sample contains microcracks that can be attributed to the temperature gradient and difference between the high-temperature left side and low-temperature right side, and the uneven cooling after welding. Hence, a post-weld shot-peening treatment is necessary for the crack closures. It is also evident that the sample that was shot-peened with high intensity (bottom row in the figures) is the roughest. This is plausible since the high-intensity shot-peening was performed with small steel balls, while the low-intensity shot-peening utilized ground glass balls. To study the roughness across zones A, B, and C, Figure 11 and Figure 12 must be viewed column-wise. It can be concluded that the heat-affected zone (region B) has the least roughness compared to the weld bead (region C) and the unaffected area (region A); shot-peening did not change the relative roughness. In addition, low-intensity shot-peening closes microcracks, as can be seen by comparing the top two images in the center column of Figure 11. High-intensity shot-peening not only closes the microcracks, but also introduces major surface deformations.

Figure 13 shows the optical approximations of the 3D surface topographies in the samples of Figure 11. This qualitative view may confirm that the heat-affected zones (the center column of the figures) have the least roughness relative to the other two regions. The last two rows also show the indentations (or dimples) resulting from the shot-peening process.

Further image analysis was performed to find the distribution of dimple size in each of the shot-peened ROIs. These results are shown in Figure 14. The number of dimples was counted for a size range from 20 to 150 micrometers. This was achieved by running a matched filter with a variable-diameter disk as a filter mask. The analysis shows that the low-intensity shot-peened ROIs (top row) do not contain dimples larger than 100 micrometers, whereas the high-intensity shot-peened samples do. Within the sample, using row-wise inspection, it is evident that the heat-affected zones in *B_Weld+Low_* and *B_Weld+High_* have the most dimples of 100-micrometer size.

The roughness was calculated for the samples shown in Figure 11 using the metrics defined in Equations (4) through (6), and plotted in Figure 15. It is evident that the shot-peening process increases the roughness of the surface. Our proposed algorithm was capable of capturing and quantifying surface roughness parameters *R_a_*, *P_p_K* and *P_v_K*. The overlapping observed in the errors bars in Figure 15 shows that our optical metrics can detect the roughness caused by shot-peening; however, they are only marginally suitable for differentiating roughness caused between high- and low-intensity shot-peening.

The high-intensity shot-peened ROIs are the roughest, which corresponds with the qualitative observations explained previously. The results also confirm that the heat-affected zones are the least rough compared to the shot-peened weld bead and unaffected zone. It is also evident that the weld-beads zone responded similarly for both high- and low-intensity shot-peening. In other words, the surface of the weld beads does not change significantly.

This was attributed to the higher hardness of the weld beads zone (region A) compared to the heat-affected zone (region B), which made it less affected by the shot-peening intensity after a certain level. Hence, it is concluded that a measurement of sub-surface hardness is necessary, as shown in Figure 16. It was observed that the surface roughness profile somehow mimics the hardness profile for both high- and low-intensity shot-peened samples, where the W shape was observed. This was attributed to the higher hardness region, which corresponded to higher hardness values; as a result, it is less affected by shot-peening surface treatments.

## 4. Conclusions

In this paper, we have described an image processing algorithm to measure the surface roughness in TIG-welded aluminum 6065-T6 alloy. Using the proposed technique, it was possible to measure the surface roughness of the weld beads, heat-affected zone, and base metal alloy for control samples (TIG-welded only), as well as for high- and low-intensity shot-peened, TIG-welded samples. Furthermore, 3D topography images were generated in a simple manner and used for qualitative analysis. Furthermore, the experimental results showed that dimple size and distribution measurements are possible for shot-peened samples. Optical line profiles were used to calculate roughness metrics to overcome the limitations of measurement equipment in the irregular weld bead area. The final metrics calculated were the averages of the whole region of interest (ROI) data, such that it covers all line profiles and delivers roughness estimates across the whole ROI rather than just a few selected lines, as is the case of laboratory measurements. Also, a blurring filter was used with a 15 × 15 Gaussian mask for convolution and it showed a smoothed image histogram, in which it is evident that the pre-blur histogram has empty intensity bins, which is attributed to the TIFF image format compression artifact compared to blurred ones. Hardness profiles showed that for all tested scenarios of welded only, high- and low-intensity shot-peened samples, softening was observed in the region of weld beads and heat-affected zones compared to base metal alloy. In addition, the heat-affected zone has the least hardness in general, and high-intensity shot-peening is harder than low intensity. Lastly, the presented optical roughness metrics are able to differentiate between shot-peened and untreated alloy surfaces as the metric averages do not lie with the variances of each other. However, this is not the case for high-intensity versus low-intensity shot-peening roughness measurement. Here, only qualitative conclusions are possible because the metric averages lie within the variances of each other.

## Figures and Tables

**Table 1:** Chemical composition of aluminum alloy Al 6061-T6.

Chemical Composition (wt %)								
Si	Fe	Cu	Mg	Mn	Ti	Zn	Cr	Al
0.8	0.7	0.4	1.2	0.15	0.15	0.25	0.35	Rem.
